# Intraoperative Blood Loss in Female Patients with Adolescent Idiopathic Scoliosis during Different Phases of the Menstrual Cycle

**DOI:** 10.1371/journal.pone.0112499

**Published:** 2014-11-25

**Authors:** Chao Li, Yang Xie, Zhikun Li, Mingyuan Yang, Xiaofei Sun, Jianping Fan, Honglei Yi Xiaodong Zhu, Chuanfeng Wang, Ming Li

**Affiliations:** 1 Department of Spine Surgery, Changhai Hospital, the Second Military Medical University, Shanghai, China; 2 Department of Orthopedics, Changhai Hospital, the Second Military Medical University, Shanghai, China; University of Oulu, Finland

## Abstract

**Background:**

The vast majority of AIS patients who require surgical intervention are women. Blood loss is a major concern during the operation.

**Methods:**

The medical records of all female AIS patients who underwent posterior correction and fusion operations using the all-pedicle screw system from January 2012 to January 2014 were reviewed. Patients with irregular menstruation; underwent osteotomy; use coagulants were excluded from the study. The remaining patients were divided into 4 groups according to the operation date in the menstrual cycle (A: premenstrual group, 24–30 d; B: follicle group, 6–11 d; C: ovulatory group, 12–17 d; D: luteal group, 18–23 d). The information of patients from the 4 groups was reviewed. The data was analyzed using analysis of variance, the Student-Newman-Keels test and Kruskal-Wallis Test.

**Results:**

A total of 161 patients were included in this study. There were 40 patients included in group A, 38 patients in group B, 41 patients in group C and 42 patients in group D. The 4 groups were matched in age (P = 0.238), body height (P = 0.291), body weight (P = 0.756), Risser sign (P = 0.576), mean curve Cobb angle (P = 0.520), and bending flexibility index (P = 0.547), the number of levels fused (P = 0.397). The activated partial thromboplastin time (P = 0.235) and prothrombin time (P = 0.074) tended to be higher in group A, but the difference was not statistically significant. The fibrinogen level was lower in group B than the other 3 groups (P = 0.039). Blood loss and normalized intraoperative blood loss (NBL) was significantly higher in group A than the other 3 groups (P<0.01).

**Conclusions:**

The hemostatic function tended to be lower in the premenstrual phase. The fibrinogen level was lowest in the mid-follicle phase. Female AIS patients tended to endure more intraoperative blood loss when the operation was performed in the premenstrual phase during the menstrual cycle.

## Introduction

Adolescent idiopathic scoliosis (AIS) is a structural, lateral, rotated curvature of the spine that arises in otherwise healthy children at or around puberty. AIS may affect 1–3% of the at-risk population when defined as a Cobb angle>10° [1]. Surgical intervention may be required when the Cobb angle is greater than 40°. For patients with a major curve greater than 30°, female/male ratio could be as high as 10∶1. The likelihood of curve progression in female patients is even higher [2]–[4]. The vast majority of AIS patients who require surgical intervention are women.

Intraoperative blood loss (IOBL) is a major concern during the operation and may lead to many complications such as hypotension, anemia, coagulopathy, infection, and the need for transfusion of large volume of blood products with associated risks [5]. It is also associated with a higher prevalence of non-neurologic complications [6]. Numerous hemostatic agents have been used to reduce the IOBL during scoliosis surgery and have demonstrated variable efficacy; however, mortality, morbidity (need for blood transfusion, rebleeding, or other complications), and medico-economic criteria have not been appropriately evaluated [5], [7]–[9].

Sex hormone variations may affect the level of blood coagulation factors, thus, influence the hemostatic function in female adolescents [10], [11]. Some studies showed that treatment with contraceptive medication may affect the hemostatic potential in women [12]–[14]. Sex hormone variations were also thought to be related to the hypercoagulable status during pregnancy [15], [16]. However, studies of hemostatic status during the different phases of the normal physiological menstrual cycle show contradictory results [10]. The current study aimed to determine if clinical hemostatic indicators and IOBL vary among female AIS patients operated in different phases of the menstrual cycle. To the best of our knowledge, no studies have addressed this issue to date.

## Methods

### Objectives

In this retrospective analysis, we investigated the effect of operation time during different phases of menstrual cycle on IOBL in female adolescent idiopathic scoliosis patients who underwent posterior correction and all pedicle screw fixation. Previous studies have shown that increase in blood sex hormone could cause blood coagulation changes. We speculate that natural hormone variation during normal menstrual cycle may affect blood coagulation too. This variation is especially meaningful considering that the majority of AIS patients who need surgical intervention are female and the large IOBL during posterior correction and fusion surgery for AIS patients.

### Participants

Female AIS Patients, who sought medical help and underwent posterior correction and all pedicle screw fixation and fusion surgery in Chinghai hospital between January 2012 and January 2014, were retrospectively reviewed. The diagnosis of AIS followed the description of Weinstein et al [1]. Other subtypes of scoliosis were excluded by medical history, physical and radiological examination. Diagnosis was made independently by three attending clinicians, and only patients who were diagnosed with AIS by all the three doctors were included in this study. Details of the patients' menstruation history including the date of last menstruation, duration of menstruation, duration of menstrual cycle and rhythm of menstrual cycle were attained from the medical record (this information was record on the medical record according to the medical record writing norm issued by Ministry of Health of China), Patients with irregular menstruation, underwent osteotomy, using coagulants or combined with other diseases that may affect the operation were excluded. The remaining patients were divided into 4 groups according to the operation date during the different phases of the menstrual cycle (A: premenstrual group, 24–30 d; B: follicle group, 6–11 d; C: ovulatory group, 12–17 d; and D: luteal group, 18–23 d).

### Follow-up Outcome Measures

Parameters, including: patient age; body weight; body height; the number of levels fused; major curve Cobb angle; major curve bending flexibility index; blood type; albumin (Alb); hemoglobin (Hg); fibrinogen; platelet count (PLT); activated partial thromboplastin time (APTT); prothrombin time (PT); thrombin time (TT); IOBL; operation date and menstrual cycle data were reviewed. Normalized intraoperative blood loss (intraoperative blood loss per level fused per kilogram, NBL) was calculated using the following equation:
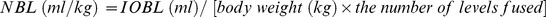



### Patients and Anthropometric Measurements

The study protocol was approved by the Institutional Review Board of the Second Military Medical University, Shanghai, China. Written informed consent was obtained from every participant. The protocols of this study have been approved by the institutional review board of Changhai Hospital.

### Statistical analysis

Data from different groups was analyzed using analysis of variance (ANOVA), the number of levels fused was analyzed by Kruskal-Wallis Test. Differences between the groups were further analyzed using the Student-Newman-Keuls (SNK) test. The data was checked for normality and equal variances. A P value less than 0.05 was considered significant for each individual test in this study. Statistical Package for Social Science software 18.0 (SPSS Inc., Chicago, IL, USA) was used to perform the statistical analysis. Graphs were drawn using GraphPad Prism 5.0 (GraphPad Software Inc., San Diego, CA, USA).

## Results

### Baseline and Perioperative Characteristics of Patients

A total of 276 female AIS patients underwent posterior correction and spinal fusion were screened. Among them, 115 patients were excluded because of: Ponte Osteotomy during the surgery (38); combined anterior approach (17); diagnosed coagulopathies (9); menstrual cycle problems (44, irregular menstrual cycle, menstrual cycle longer than 30 days or shorter than 28 days, before menarche); or intraoperative hemostasis (43). The remaining 161 patients were reviewed. There were 40 patients included in group A, 38 patients in group B, 41 patients in group C, 42 patients in group D. ANOVA showed that the 4 groups were matched in age (P = 0.246), body height (P = 0.359), body weight (P = 0.348), Risser sign (P = 0.628), mean curve Cobb angle (P = 0.596), bending Cobb (P = 0.993), bending flexibility index (P = 0.849). Kruskal-Wallis Test showed there was no difference in the number of levels fused (P = 0.497). The APTT (P = 0.168) and PT (P = 0.107) tended to be higher in group A than the other groups; however, the difference was not statistically significant. There were no significant differences in Hg (P = 0.875), PLT (P = 0.517), Alb (P = 0.247), and TT (P = 0.949) between the groups. There were significant differences in fibrinogen (P = 0.039), IOBL (P<0.01), and NBL (P<0.01) between the groups. The details of the 4 groups analyzed by ANOVA are shown in Table 1.

**Table 1:** Clinical characteristics of the patients.

Variable	Group A	Group B	Group C	Group D	Mean	P
Number	40	38	41	42		
Age (y)	15.22±2.03	14.57±1.66	14.66±1.53	15.13±1.74	14.90	**0.246**
Height (cm)	158.55±4.71	159.42±4.18	157.71±3.67	158.80±4.64	158.60	**0.359**
Weight (kg)	47.04±6.37	48.95±5.66	46.88±5.86	48.27±5.88	47.76	**0.348**
Risser sign	3.60±1.19	3.45±1.03	3.38±1.19	3.29±1.23	3.43	**0.682**
Major curve (°)	51.60±11.99	49.34±10.27	48.48±8.14	49.54±11.67	49.73	**0.596**
Bending cobb (°)	13.28±7.46	13.39±6.74	13.10±6.55	13.00±5.59	13.19	**0.993**
B-Flexibility index	0.75±0.10	0.73±0.12	0.73±0.12	0.74±0.08	0.74	**0.849**
Alb (g/L)	41.45±2.09	41.50±2.05	40.83±2.19	40.78±1.97	41.13	**0.247**
Hg (g/L)	123.98±7.04	125.08±8.67	125.31±8.72	125.17±7.89	124.89	**0.875**
PLT	215.85±35.67	221.37±38.53	222.21±52.09	230.46±46.79	222.53	**0.517**
APTT (s)	38.34±1.90	37.14±2.39	37.55±2.97	37.54±2.19	37.65	**0.168**
PT (s)	13.58±0.41	13.39±0.37	13.37±0.47	13.36±0.51	13.42	**0.107**
Fibrinogen (g/L)	2.62±0.44	2.35±0.47	2.57±0.47	2.60±0.43	2.54	**0.039**
Thrombin time (s)	16.90±1.67	16.97±1.70	16.87±1.30	16.77±1.50	16.88	**0.949**
The number of levels fused	10.65±1.67	10.79±1.70	10.64±2.08	10.15±2.22	10.55	**0.497**
IOBL (ml)	1078.75±295.28	952.11±194.27	863.81±201.77	847.80±266.03	933.98	**<0.01**
NBL (ml/f*kg)	2.17±0.46	1.83±0.34	1.77±0.34	1.76±0.40	1.87	**<0.01**

**Figure 1:**
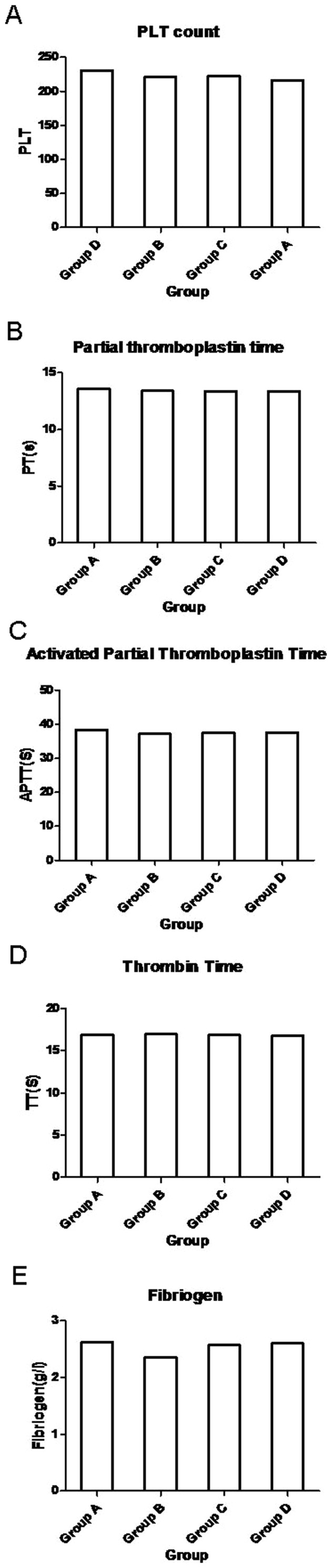
Variations of clinical coagulate indicators between groups. There were no difference between groups in PLT count(A), PT(B), APTT(C), TT(D); The Fibrigen level in group B was significant lower than other 3 groups(E).

**Figure 2:**
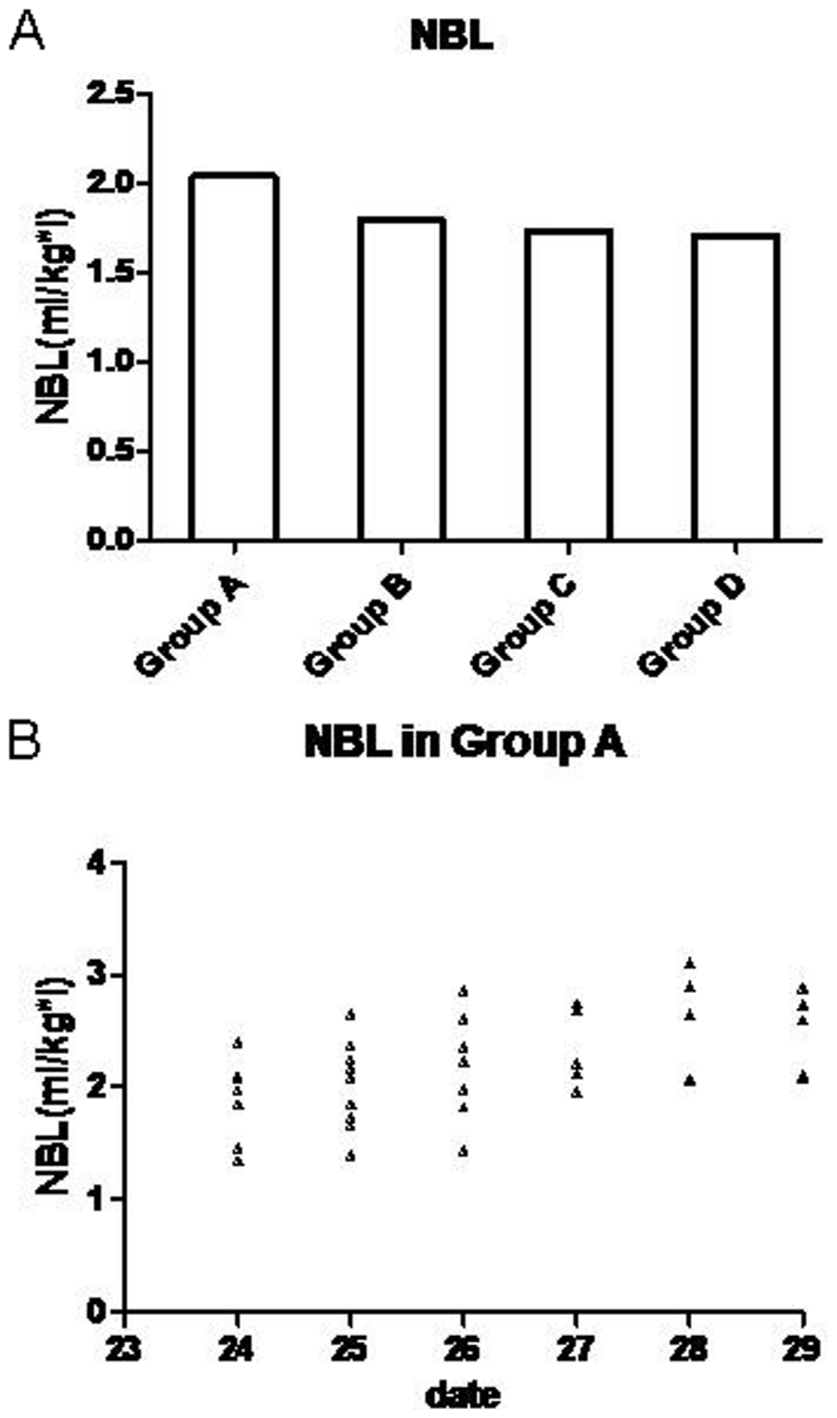
Average NBL between different groups and scatter diagram of NBL in group A. NBL between groups (A) show NBL was significantly higher in group A than the other 3 groups, There was no difference between other 3 groups. Scatter diagram of NBL (B) in group A show the NBL has a tendency to increase during the last days of the menstrual cycle and achieve a peak during 1–2 days before Menstruation.

The SNK test (fibrinogen, blood loss, NBL) showed that the IOBL and NBL were significantly higher in group A than in other groups; there was no observed difference between groups B, C, and D. Fibrinogen was significantly lower in group B than groups A, C, and D; there was no observed difference between groups A, C, and D. Tables 2, 3, and 4 show data analyzed using the SNK test regarding fibrinogen, blood loss, and NBL. The fluctuations of the clinical coagulation test are shown in Figures 1(A–E). Figure 2 shows the differences in NBL between each group and the scatter point of NBL in group A according to the day of the menstrual cycle; the NBL tended to be higher among the subjects who underwent surgery in the last days of the menstrual cycle.

**Table 2:** Student-Newman-Keuls test: Fibrinogen.

Group	N	Subset for alpha = 0.05	
		1	2
B	38	2.354211	
C	42		2.569048
D	41		2.602927
A	40		2.618750
Sig.		1.000	.876

Table 2 shows the Fibrinogen level was NBLsignificant lower in group B.

**Table 3:** Student-Newman-Keuls test: NBL.

Team	N	Subset for alpha = 0.05	
		1	2
D	42	1.755241	
C	41	1.769632	
B	38	1.829504	
A	40		2.167856
Sig.		0.666477	1.000

Table 3 shows the NBL was significant higher in group A.

**Table 4:** Student-Newman-Keuls test: Blood loss.

Team	N	Subset for alpha = 0.05	
		1	2
D	41	847.80	
C	42	863.81	
B	38	952.11	
A	40		1078.75
Sig.		0.136	1.000

The means for groups in homogeneous subsets are displayed.

a. Uses harmonic mean sample size  = 40.195.

b. The group sizes are unequal. The harmonic mean of the group sizes is used. Type I error levels are not guaranteed.

Table 4 shows the Blood loss was significant higher in group A.

## Discussion

Posterior correction with multilevel spinal fusion (PCSF) has been proven to be the most effective way to treat AIS [1], [17], [18]. With a long fusion range, PCSF is associated with significant IOBL normally ranging from 750 to 1500 mL [19]. This was confirmed by our study, with a mean blood loss of 933.73 mL (500–2000 mL). Both increased IOBL and blood transfusion are associated with hypotension, anemia, coagulopathy, infection, allergic reactions, acute or delayed immune hemolytic reactions, iron overload, and graft-versus-host disease [5], [6], [20]. Numerous hemostatic agents have been used to reduce the IOBL during scoliosis surgery and have demonstrated variable efficacy without enough consideration about complications [5], [7]–[9].

A significant finding in our study was that patients in group A experienced significantly higher IOBL (total: 1078.25±295.28 mL; NBL: 2.04±0.35 mL/kg) than those in group B (total: 952.11±194.27 mL; NBL: 1.80±0.30 (mL/kg*l), group C (total: 864.81±201.77 mL; NBL: 1.73±0.27 mL/kg*l), and group D (total: 846.83±266.63 mL; NBL: 1.71±0.34 (mL/kg*l). Even in group A, the NBL of patients tended to increase as the operation date approached menstruation. This indicates that patients who undergo surgery before menstruation may experience more blood loss. The differences between the groups may be attributed to the hormone-induced hemostatic function change. Van Roojen et al [21] studied the blood hemostatic factors of 11 women before and after taking emergency hormonal contraception (which could rapidly increase the blood estrogen level). They found the plasma concentrations of free protein S, F1+2, and Factor VIIa and the APTT-based APCsr changed toward a more precoagulant state. Ibrahimi et al [11] studied 49 women who received oral contraceptive and found increased concentrations of fibrinogen and factor VIII as well as decreased PT and APTT after treatment. Most of the studies show that women taking estrogen/progesterone will have an increased level of factor VII, factor X, factor XII, and fibrinogen. Furthermore, both the level and activity of antithrombin antigen were decreased while the levels of factor V and factor VIII remained unchanged [22]. These studies show a positive association between oral estrogen and hemostatic ability.

However, studies on variations in hemostatic factors during the normal menstrual cycle are rare and show contradictory results. Miller et al [23] conducted a cross-sectional study of 175 women and found that the levels of Von Willebrand factor (VWF) and factor VIII were lowest during menstruation. Similar results were confirmed by some studies [24]–[26], while other studies reported that there were no cyclic variations in VWF, factor VIII, and factor XI during the menstrual cycle [24], [27]–[30]. Cederblad et al [31] studied 30 normal women whose blood samples were taken on 6 occasions: day 1, 2 and 3 of menstruation; day 5–9 (follicular phase); day 12–16 (around ovulation); and day 19–23 (luteal phase). They found that the concentration of factor II–VII–X was lowest during menstruation. Bolis et al [32] reported a significant decrease of factor XIII during the periovulatory phase in a study of 10 women. The present study shows that both APTT and PT were higher in group A than in the other 3 groups (Table 1; Figure 1, B and C), but the difference was not statistically significant (P = 0.168, P = 0.107) and still confined within the normal range. However, this difference may have been induced by the changes of hemostatic factors, which then caused the difference in IOBL between the groups.

Another finding of this study was that the fibrinogen level was lowest in the follicle phase (group B: follicle group, Figure 1 E, P = 0.039). Several studies reported on the variations of fibrinogen levels during the menstrual cycle. Six of these studies reported the lowest levels of fibrinogen during the follicular or mid-cycle phase [24], [27], [29], [33]–[35], 2 reported the lowest levels during luteal phase [36], [37], while other studies reported no cyclic variation [28], [38]–[40]. We speculate that the reason for this may be the consumption during menstruation and the relatively low level of sex hormone during menstruation, since oral estrogen increases fibrinogen concentration in blood [41], [42].

We normalized the IOBL by dividing blood loss by number of levels fused and by patients' weight. The number of levels fused has been reported to be a predictor of IOBL in scoliosis surgery [43], [44]. In the present study, body weight significantly varied between patients; this was caused by the individual differences in the development stages during puberty. This method was also used by Amit et al [45] when comparing the blood loss between different types of scoliosis surgery; the method proved to be an effective way to eliminate the effect of the number of levels fused and body weight.

One of the concerns of our study was its retrospective design. We relied on the IOBL noted on the medical records when collecting the data. However, the estimation of IOBL in scoliosis surgery was performed by 2 senior anesthesiologists in our hospital, while following strict criteria. In other words, all the AIS patients were evaluated by the same group of physicians using the same criteria, thus, individual error was minimized. Another concern of the study might be lack the data of patients from 1^st^–5^th^ days of menstrual cycle. Actually, we rarely perform the posterior correction and fusion surgery during menstruation. There were two reasons that we did not perform the surgery during menstruation. Firstly, the posterior correction and fusion surgery was featured by its large IOBL, but during menstruation the patients were experiencing fundamental blood loss. Secondly, we think menstruation was a kind of injury to the endometrium of the female AIS patient. We want to do our best to avoid these shortcomings since the surgery we are going to perform is a selective operation.

Our study had several strengths. First, our data was collected from a single institution. All the treatments followed a uniform procedure, and all the evaluations were performed using the same criteria. We confined our review time to 2 years to avoid confounding factors such as changes in the surgeon's surgical skills, improvement of equipment, etc. This minimized the system error of this study. Second, we eliminated any factors that may have affected our study, such as age over 18 years (patients may have a rigid curve as their age increases thus increase the IOBL), combined anterior and posterior approach, use of antifibrinolytic medication before or during surgery, and use of osteotomy during surgery. Finally, rather than only dividing the patients into follicle and luteal groups as in previous clinical and basic studies related to this field [25], [27], [29], [38], [39], [46], [47], we divided our subjects into 4 groups which included perimenstrual days; this made our study more comprehensive and allowed us to find that patients who underwent surgery during the premenstrual phase tended to endure more IOBL. As a selective operation with relatively high IOBL, in female AIS patients, it may be more plausible to perform the correction and fusion surgery at a different time than premenstrual days during the menstrual cycle.

## Conclusions

Female AIS patients tended to endure more IOBL when the operation was performed in the premenstrual phase of the menstrual cycle. The hemostatic function tended to be lower in the premenstrual phase; however, statistical significance was not reached. The fibrinogen level was lowest during the mid-follicle phase.
